# Three Critical Issues to Consider Before Implementing a New Genome-Cohort Study in Japan

**DOI:** 10.2188/jea.JE20100177

**Published:** 2011-03-05

**Authors:** Akiko Tamakoshi, Kenji Matsui, Keiko Sato, Tohru Masui, Eiji Maruyama

**Affiliations:** 1 Aichi Medical University School of Medicine, Aichi, Japan; 2 The University of Toyama Center for Clinical Bioethics, Toyama, Japan; 3 Kyoto University Graduate School of Medicine, Kyoto, Japan; 4 National Institute of Biomedical Innovation, Osaka, Japan; 5 Kobe University School of Law, Kobe, Japan

## INTRODUCTION

The Council for Science and Technology recently announced a new genome-cohort project—scheduled to start in 2013—for the promotion of research in life science innovation in Japan.^1^ Additionally, a large ongoing project to build biobanks at 6 national centers will provide a foundation for future cohort studies.^2^ Such studies investigate the pathogenesis of disease by following large numbers of participants over an extended period of time and comparing factors such as lifestyle, clinical characteristics, biomarkers, and gene polymorphisms. From the perspective of disease prevention, it is important to investigate gene–environment interactions in humans. For this purpose, we need genome-cohort studies. Based on our experience in the development of a large-scale collaborative cohort project, we suggest three key issues that need to be addressed when planning new genome-cohort studies.

## I. PLANNING AND DATA COLLECTION

Based on our previous experience in managing cohort studies, we believe it remains a challenge to (1) examine the impact on disease of uncommon or combined lifestyle factors, (2) investigate risk factors of rare diseases, and (3) analyze specific subgroups, such as male nonsmokers, even with a cohort study that followed 100 000 subjects for more than 10 years. For these reasons, the principal investigators of existing cohorts are currently trying to collaborate, so as to increase the number of study subjects and analytic power of studies. For example, in Japan, the Research Group for Development and Evaluation of Cancer Prevention Strategies has published results from a pooled analysis of large-scale cohort studies in cancer research,^3^ and the EPOCH Study group has similarly reported pooled results in cardiovascular research.^4^ However, one hurdle in collaboration is that each cohort uses a different questionnaire. We have found that even for seemingly straightforward variables, such as smoking habit, it is not easy to integrate data. Moreover, different methods of biospecimen collection and storage make the interpretation of measurement results difficult. Also, the scope of consent obtained from participants often differs with respect to study purpose, limitations on collaborative use of biospecimens, and expiration date. Accordingly, when planning a new genome-cohort study, it is advisable to develop a standardized system under which data from different cohort studies can be combined, if necessary. During a study follow-up period, it may prove necessary to collect additional or different information or develop new methods of analysis. To account for such changes in time (eg, technological development and long-term changes) and scope (methodological development and expansion to a collaborative study) after study initiation, full consideration must be given not only to standardizing questionnaires and methods of biospecimen collection, but also to ensuring that the scope and nature of the consent form used at the start of a study encompasses, to the greatest possible extent, any potential future transfer, sharing, and secondary use of donated samples and data.

## II. RESEARCH FUNDING AND EVALUATION

In a cohort study, a large number of subjects are recruited and followed over time. During recruitment, we generally inform the subjects of the research plan, obtain their consent, ask them to complete a questionnaire, record the results of their physical examination, and obtain biospecimens from them. During the follow-up phase, we need to monitor disease incidence or mortality among the subjects. Generally, the costs of the recruitment phase are known in advance; however, the difficulty and expense of following all participants properly and completely tend to be underestimated. Most cohort studies in Japan are funded by government ministries. One characteristic of this funding is that a common system is used to evaluate research outcomes every few years based on short-term study results, without any consideration of the differences in the fields of study or nature of the research. The success of a research outcome is typically based on the number of publications and/or patents acquired; however, this evaluation system is not suitable for cohort studies, which require a considerable period of time before results are published. Moreover, if funding is gradually reduced or suddenly altered during the study period, the cohort study might no longer be feasible, as a budget forecast is necessary to run the study. One new cohort study, the Japan Environment and Children’s Study, which started in 2010,^5^ is an exception, as its essential funds have been promised for a 16-year period. To successfully complete large-scale genome-cohort studies in Japan, stakeholders must revise their research evaluation systems and budgeting processes by adopting a long-term view.

## III. COORDINATING CENTERS AND STAFF CAREER PATHS

To carry out a large-scale cohort study, various activities, such as preparation, recruitment, management, and safekeeping of documents and biospecimens, need to run in parallel and in a timely manner. This requires sound administrative functioning in the research coordinating center. Current cohort studies in Japan are mainly conducted by staff in one department at a university or research institution. However, because publication of cohort study results takes years, most researchers need to pursue other research to receive favorable evaluations of their research performance. Researchers simultaneously involved in other studies often face difficulties in taking full responsibility for running a cohort study. Such a situation can cause delays in dealing with difficulties arising during the study or loss of participant trust in the researchers. If, instead, a researcher were to engage exclusively in a cohort study, his/her career path would likely suffer because the research evaluation systems used in Japan do not consider contributions to cohort study management as scientific achievement. This also applies to other study personnel, such as research associates and research coordinators. Without research associates, it is impossible to properly administer a large-scale cohort study. However, the positions of such staff are not guaranteed because many are employed with unstable funds as part-time or short-term employees and opportunities for career advancement are rarely available to them. In addition, they may need to change jobs every few years when funding ends. Therefore, even if such staff master the necessary skills and become experts in their field of study, they often have to leave their job before they are able to fully demonstrate their scientific capabilities. This represents a hugely unproductive use of valuable human resources, as well as wasteful spending on personnel training costs. International cohort studies are currently implemented with massive lifestyle and environmental information and biospecimen databases and are constantly incorporating the newest methodological techniques. In such a highly technologically oriented era, with vast amounts of available information, successfully managing a large-scale genome-cohort study in Japan that can compete with international cohort studies is not an easy task; thus, we clearly must improve the capabilities of coordinating centers. Therefore, we urgently need to establish fully functional coordinating centers, revise assessment systems to evaluate personnel performance fairly, and create a support system to help with the career advancement of research staff.

## CONCLUSION

Scientific evidence on health is collected in epidemiologic studies. To steadily accumulate accurate scientific evidence, new genome-cohort studies should be executed by addressing the three issues discussed in this article. It is also necessary for researchers and research associates to share their skills and know-how throughout the study period to complete and successfully transfer the study to the next generation.

## References

[1] Science and Technology Policy. http://www8.cao.go.jp/cstp/budget/action.html [accessed on Nov 19, 2010].

[2] Press conference on October 20, 2010 by Dr. Takamasa Kayama, Chief Director of National Cancer Center.

[3] Mizoue T , Inoue M , Wakai K , Nagata C , Shimazu T , Tsuji I , ; Research Group for Development and Evaluation of Cancer Prevention Strategies in Japan Alcohol drinking and colorectal cancer in Japanese: a pooled analysis of results from five cohort studies . Am J Epidemiol. 2008;167:1397–406 10.1093/aje/kwn073 18420544

[4] Murakami Y , Hozawa A , Okamura T , Ueshima H ; Evidence for Cardiovascular Prevention From Observational Cohorts in Japan Research Group (EPOCH-JAPAN) Relation of blood pressure and all-cause mortality in 180 000 Japanese participants; Pooled analysis of 13 cohort studies . Hypertension. 2008;51:1483–91 10.1161/HYPERTENSIONAHA.107.102459 18443234

[5] Japan Environment and Children’s Study. http://www.env.go.jp/chemi/ceh/index.html [accessed Nov 19, 2010].

